# French Language Online Cognitive Behavioral Therapy for Insomnia Disorder: A Randomized Controlled Trial

**DOI:** 10.3389/fneur.2019.01273

**Published:** 2019-12-05

**Authors:** Régis Lopez, Elisa Evangelista, Lucie Barateau, Sofiene Chenini, Adriana Bosco, Michel Billiard, Anne-Dominique Bonte, Séverine Béziat, Isabelle Jaussent, Yves Dauvilliers

**Affiliations:** ^1^Centre National de Référence Narcolepsie Hypersomnies, Unité des Troubles du Sommeil, Département de Neurologie, Hôpital Gui-de-Chauliac, Montpellier, France; ^2^Inserm U1061, Montpellier, France; ^3^Université de Montpellier, Montpellier, France; ^4^Département de Neurologie, Hôpital Gui-de-Chauliac, Montpellier, France; ^5^Meta-coaching, Paris, France

**Keywords:** insomnia, cognitive-behavioral therapy, internet-based intervention, adults, randomized controlled trial

## Abstract

**Background:** Despite cognitive-behavioral therapy for insomnia (CBT-I) being the recommended treatment for insomnia disorder, its access remains very limited. Automated Internet-delivered CBT-I (eCBT-I) is an emerging cost-effective strategy for adults with insomnia, however no such program is currently available in French Language. We evaluated a French-speaking, eCBT-I intervention to improve insomnia disorder in comparison to minimal psychoeducation therapy (mPT).

**Methods:** Forty-six adults with insomnia disorder were randomly allocated to eCBT-I or mPT. The eCBT-I program consisted of seven sessions that delivered the typical components of CBT-I during 12 weeks. The mPT provided structured and non-tailored information about sleep and insomnia during a 1 h session. Insomnia severity Index (ISI, primary outcome), measures of fatigue, sleepiness, anxiety, depressive symptoms and quality of life were collected at baseline and endpoint. Electronic sleep diaries were completed over 2 week periods pre- and post-intervention.

**Results:** Compared to mPT, eCBT-I resulted in greater decrease in ISI scores between baseline and endpoint. Sleep diaries parameters improved in both groups, with a greater improvement in the eCBT-I group. Patients allocated to eCBT-I group also improved depressive, fatigue, anxiety symptoms, and quality of life. Among patients with CNS-active drug at baseline, 91.7% reduced or stopped their hypnotic medication, and 16.7% in the mPT group.

**Conclusions:** The present eCBT-I program seems feasible, acceptable and effective in reducing insomnia severity and insomnia-related functional outcomes in this small clinically-derived population. Given the high prevalence of insomnia, our data are supportive of the use of such program as an effective alternative to treat insomnia in daily clinical practice in French speaking countries.

## Introduction

Insomnia disorder is very prevalent and affects 6–15% of the general population according to various definitions, severity and phenotypes (1). Insomnia is characterized by dissatisfaction with sleep quality or quantity with difficulty initiating or maintaining sleep, associated with daytime dysfunction and deterioration of the quality of life (2, 3). Insomnia disorder is often chronic, and its persistence may be associated with psychiatric and cardiovascular diseases (4), and even mortality (5).

According to international recommendations, cognitive behavioral therapy for insomnia (CBT-I) should be the first-line treatment of persistent insomnia disorder, instead of hypnotic benzodiazepines or Z-drugs or sedative antidepressants with mixed long-term results, frequent side effects, dependence, and tolerance over time (6). Several meta-analyses have highlighted the efficacy of CBT-I improving both sleep and functional outcomes, with effects being maintained for years (7, 8). Traditionally, CBT-I is delivered face-to-face, individually or in groups, but with limited access due to the insufficient availability of trained therapists, and its high cost. Insomnia remains an unrecognized and undertreated condition despite its important societal cost, high level of use of health care, and impaired work productivity and absenteeism (9, 10).

To overcome the limitation of CBT-I accessibility to the general population, promising methods were proposed to deliver automated CBT-I program with self-help materials (11), and more recently over the Internet with several existing commercial CBT platforms (i.e., Sleepio, SHUTi, Restore…). Since the first randomized controlled trial (RCT) evaluating online CBT-I (eCBT-I) (12), several programs have been evaluated showing improvements in several sleep outcomes, including self-assessed insomnia severity, total sleep time (TST), sleep efficiency (SE), sleep latency (SL), wake after sleep onset (WASO) and the number of nighttime awakenings (NWAK). Two recent meta-analyses of RCT of eCBT-I have been recently conducted and found improvement in both severity of insomnia and sleep efficiency (13, 14). These changes were comparable to improvement reported after either face-to-face delivered CBT-I or pharmacotherapy for insomnia (13). Some studies also highlighted the benefit of behavioral interventions to attenuate withdrawal symptoms and prevent insomnia relapse (8, 15–17), however contrasting results were obtained using eCBT-I in achieving hypnotic tapering (12, 18–25).

Nowadays, eCBT-I is accepted as an effective treatment for adults with chronic insomnia and as a cost-effective strategy in health care (26, 27). Although the French language is the sixth most spoken in the world, with 220 million speakers, including France, but also other predominantly French-speaking areas such as Belgium, Switzerland, Quebec and some African countries, to our knowledge, no eCBT-I program was currently available.

The aim of this study was to compare the efficacy of a French-speaking automated eCBT-I program in a small population of clinically-derived population of patients with persistent insomnia disorder in comparison to an active control intervention.

## Methods

### Study Design

The study was a parallel-group, RCT with two treatment arms: (1) eCBT-I program and (2) minimal psychoeducation therapy (mPT: placebo). The randomization sequence was computer-generated using random blocks in an order unknown by the investigators.

### Participants

Participants were recruited from the outpatient clinic for sleep disorders of the Gui-de-Chauliac Hospital, Montpellier, France, from July 2015 to February 2016. All patients underwent a structured interview with experimented clinicians to assess the presence of insomnia disorder, its characteristics (insomnia subtype, duration, frequency, and functional impairment) and severity using the insomnia severity scale (ISI), and potential comorbidities. They also completed an electronic sleep diary for 2 weeks before the randomization. Eligible participants were men and women, aged 18–64 years, who met the Diagnostic and Statistical Manual of Mental Disorders, Fifth Edition (DSM-5) criteria for insomnia disorder (2). Participants were also required to have a sleep efficiency ≤79% based on two-week sleep diary and an ISI score >14. Patients were excluded in presence of comorbid sleep disorders (sleep apnea syndrome clinically defined, restless legs syndrome at least twice a week), clinically significant psychiatric (mood, anxiety, psychotic, substance use or post-traumatic stress disorder) or medical disorders (chronic pain, respiratory, cardiovascular disease).

Central nervous system (CNS)-active drugs prescribed for insomnia (i.e., hypnotic, anxiolytic or low doses antidepressants) were accepted, however patients with more than two different CNS-active medications intake and those taking antipsychotic, opioid, antiepileptic or antiparkinsonian agents were not included.

All patients gave written consent to take part in the study. The study was approved by the local ethics committee (CPP Sud Méditerranée I, France, Number 2014-A01796-41; ClinicalTrials.gov; identifier NCT02539862).

### Procedures

Patients assigned to eCBT-I group received a 12 week automated program divided in seven sessions. The eCBT-I program was conceived by MB and ADB, and reviewed by RL. Patients ran the first session with the following components: self-assessment questionnaires about their sleep habit and complaints, followed by quiz testing their knowledge about sleep and insomnia with real time feedback. During the second session, the sleep restriction protocol was started. During the third session, behavioral therapy and stimulus control techniques were implemented. During the fourth session, three relaxation techniques (progressive muscle relaxation, imagery rehearsal and mindfulness-based approaches) were proposed and patients were invited to choose one and practice. During the fifth and sixth session, the cognitive component of the therapy was introduced with patients being invited to answer questions related to insomnia beliefs, with an automated personalized feedback to restructure dysfunctional thinking and to reduce evening ruminations. The last session consisted in revising the entire program and gave information and advices to prevent the relapse.

Among all sessions, structured information related to normal sleep, normal daytime functioning, insomnia mechanisms, hypnotic treatments, sleep hygiene rules were progressively delivered. On a daily basis, patients completed online a sleep diary in the morning to complete information on the past night, and in the evening to provide information on the daytime functioning. Based on the data collected, an automatic algorithm gave appropriate feedback to help the patients analyzing their progression and to adequately adapt the behavioral instructions. Another challenge was to ensure adherence to behavioral instructions especially when targeted to sleep restriction. Motivational advices with reward/incentive tools have been implemented all along the program. They included challenges to be accomplished within 5 to 14 days, classified into four different categories (behavioral, emotional, mindfulness, health) with habit tracking systems. Successful challenges allowed participants to earn points that unlock rewards.

Patient randomized to the control arm had a face-to-face 1 h single session of insomnia psychoeducation therapy that included structured information on normal sleep, sleep hygiene, insomnia mechanisms, and pharmacological treatment, without any behavioral instruction given to the patient.

### Outcomes

The primary efficacy outcome measure was the change in ISI from baseline to endpoint (2 weeks after the end of intervention) between the two groups. ISI, a 7-item self-report questionnaire assessing insomnia symptoms including the degree of resultant distress, was completed by all participants at baseline and endpoint. A cut-off score of 14 indicates clinically relevant insomnia while 21 indicates severe insomnia.

Secondary nighttime efficacy outcome measures were changes from baseline to endpoint between the two groups on TST, SE, SOL, WASO, NWAK assessed from sleep diaries. Patients completed an electronic sleep diary for 2 weeks before the randomization and at the end of the intervention. The electronic sleep diaries completed by participants assigned to the eCBT-I arm within the program were not used for this study. Sleep efficiency was calculated as the ratio of TST to time in bed (TIB), multiplied by 100 to yield a percentage. Information about SOL, WASO and NWAK was also retrieved from sleep diaries. Daytime efficacy outcomes included fatigue, anxiety, depressive and daytime sleepiness symptoms and quality of life assessed on validated questionnaires from baseline to endpoints. Patients completed the questionnaires twice, at baseline and endpoint to measure symptoms of sleepiness (Epworth sleepiness scale—ESS), fatigue (14-items Chalder Fatigue Scale—CFS), depression (21-items Beck depressive inventory—BDI), anxiety (State Trait Anxiety Inventory Form Y—STAI). The EuroQol group questionnaire EQ-5D is a simple instrument that provides a generic measure of health outcomes for a wide range of health conditions. It consists of two sections, the EQ-5D descriptive section and the visual analogic scale (VAS). The descriptive section assesses five dimensions of health-related quality of life (Mobility, Self-Care, Usual Activities, Pain/Discomfort, and Anxiety/Depression), each described by three response levels (no, some or extreme problems). An utility score can be calculated from individual descriptive responses. The EQ-5D VAS scores are anchored on 100 as the best imaginable health and 0 as the worst (28–32). CNS-active drugs, number, classes and doses were recorded in the two groups at both evaluations.

### Power Calculation

Statistical power was based on a sample of patients with chronic insomnia (33) with a total mean score of ISI equaled to 16.4 (SD = 3.8). Assuming that this mean would be unchanged at the end of trial for the mPT group, 18 patients per group were required to show a significant mean difference between the two groups equal to 4.23 for ISI total score with a 90% statistic power, a 2-sided 2-sample *t*-test with a significance level of 0.05.

### Statistical Analyses

Efficacy analyses were based on the modified intention-to-treat population (mITT). The primary and secondary outcomes were analyzed using the modified Full Analysis Set (mFAS), including all subjects who had one baseline and one post-baseline evaluation. The characteristics of the study population were described using the median [interquartile range (IQR)] for quantitative variables, and number and percentage for categorical variables. Chi-square or Fisher's exact tests were used to compare categorical variables between the two groups, and Mann-Whitney U test for continuous variables. Changes in each outcome between baseline and endpoint were compared between the groups using Mann-Whitney U test. For the between-group differences, 95% Confidence Intervals (CI) were constructed. To compare differences between baseline and endpoints within group, Wilcoxon signed-rank tests were used. The agreement between the two periods was evaluated with the Cohen's Kappa coefficient for dichotomous variables. The statistical analysis was done by an independent external statistician. Statistical significance was set at *p* < 0.05. Statistical analyses were performed with SAS version 9.4.

## Results

**Figure 1:**
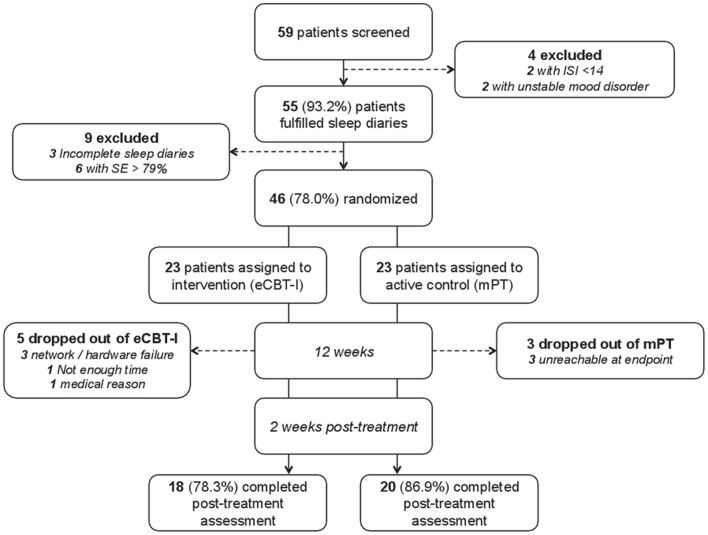
Study flowchart. SE, Sleep Efficiency; ISI, Insomnia Severity Index; eCBT-I, Online Cognitive Behavioral Therapy for Insomnia; mPT, Minimal Psychoeducation.

Fifty-nine patients were screened with insomnia disorder, of whom 13 were excluded due to not fully meeting eligibility criteria (Figure 1). The remaining 46 patients [34 females, median age 45.49 years (11.00)] were randomized to receive the study treatment: 23 were assigned to the eCBT-I and 23 to the mPT. Baseline characteristics, severity of insomnia, and rates of patients under CNS-active drug were not different between the two randomized arms (Table 1). At baseline, median BMI was 21.74 kg/m^2^ (4.92) with 11 patients being overweight. The median duration of insomnia complaints was 10.99 years (14.99). Nineteen patients had difficulties in initiating sleep, 32 in maintaining sleep and 37 reported early-morning awakenings with 33 patients having at least two symptoms. Sleep difficulties occurred with a median of 5.5 times per week (3.0). The median ISI score was 19.00 (4), with 11 patients having severe insomnia complaints. The sleep diaries of the first 2 weeks showed a median sleep efficiency of 66.67% (23.49). Thirty patients (65.2%) were taking at least one CNS-active drug, all on a daily basis.

**Table 1:** Demographic and clinical characteristics of participants randomized to Minimal psychoeducation therapy (mPT) vs. Online cognitive and behavioral therapy (eCBT-I).

	**mPT group** ***N*** **= 23**		**eCBT-I group** ***N*** **= 23**		
	***n***	**%**	***n***	**%**	***p*-value**
**Baseline demographic characteristics**					
Gender (female)	15	65.22	19	82.61	0.18
Age (years) ^(1)^	45.00 (13.00)		46.00 (11.00)		0.93
BMI (kg/m^2^) ^(1)^	21.87 (4.90)		21.48 (6.35)		0.99
BMI > 25 kg/m^2^	5	22.73	6	26.09	0.79
**Insomnia and related-characteristics during the randomization phase**					
Difficulty initiating sleep (yes)	11	47.83	8	34.78	0.37
Difficulty maintaining sleep (yes)	17	73.91	15	65.22	0.52
Early morning awakening (yes)	20	86.96	17	73.91	0.26
Insomnia disorder duration (years) ^(1)^	11.00 (14.00)		11.00 (15.00)		0.71
Frequency of insomnia (day per week) ^(1)^	5.00 (4.00)		7.00 (2.00)		0.12
At least one CNS-active medication (yes)	13	56.52	17	73.91	0.22
ISI score ^(1)^	18.00 (6.00)		20.00 (3.00)		0.11
ISI score >21	6	26.09	5	21.74	0.73
TIB (min) ^(1)^	487.14 (82.50)		513.93 (53.57)		0.28
TST (min) ^(1)^	278.21 (117.83)		296.07 (113.21)		0.48
SE ^(1)^	0.60 (0.22)		0.58 (0.22)		0.74
Sleep onset latency (min) ^(1)^	33.93 (33.57)		52.86 (63.93)		0.08
Number of awakenings ^(1)^	1.00 (0.81)		1.14 (1.07)		0.63
Wake after sleep onset (min) ^(1)^	36.43 (36.90)		36.43 (46.79)		0.87
ESS score ^(1)^	9.00 (6.00)		10.00 (7.00)		0.52
ESS > 10	7	31.82	10	43.48	0.42
BDI score ^(1)^	13.00 (11.00)		16.00 (15.00)		0.25
BDI score ≥14	11	47.83	14	60.87	0.37
EQ-5D utility score ^(1)^	0.78 (0.31)		0.69 (0.12)		0.03
EQ-5D VAS ^(1)^	60.00 (22.50)		60.00 (25.00)		0.56
STAI-E ^(1)^	34.00 (15.00)		40.00 (16.00)		0.29
CFS physical score ^(1)^	4.00 (3.00)		5.00 (2.00)		0.11

(1) *Continuous variables were expressed as median (interquartile range)*.

Eight patients prematurely discontinued the trial (5 in eCBT-I group and 3 in mPT group) (see details, Figure 1). The mITT population consisted of 38 patients: 18 patients [15 females, median age 46.50 years (11.00)] in the eCBT-I group and 20 patients [14 females, median age 45.99 years (9.50)].

Concerning the primary outcome, a significant between-group difference was found for changes in ISI between baseline and endpoint in the mITT population [eCBT-I group: −9 (7.00)] vs. mPT group −3.00 (7.00), *p* = 0.0004) (Table 2). Results remained significant in the ITT population (eCBT-I group: −8 (10.00) vs. mPT group: −3.00 (8.00), *p* = 0.004). ISI improved in both groups, with a median relative diminution of −43.91% (35.59) and −19.38% (39.08) in the eCBT-I and the mPT groups from the mITT population. Additional analyses were performed by subgroups of patients, according to their age, gender, and baseline severity (Table 3). We found significant between-group differences for ISI score with improvement in patients aged <45.5 years old, female, and in patients with baseline ISI < 19 and BDI ≥14 in the CBT-I group compared to mPT group.

**Table 2:** Effects of online cognitive and behavioral therapy (eCBT-I) compared to Minimal psychoeducation therapy (mPT) on sleep diaries parameters and self-report functional outcomes in the mITT population.

	**mPT Group (*****N*** **= 20)**				**eCBT-I Group (*****n*** **= 18)**					
	**Baseline**	**Post-treatment**	**Within-Group Difference^(1)^**		**Baseline**	**Post-treatment**	**Within-Group Difference^(1)^**		**Between-Group Difference ^(2)^**	
**Variable**	**Median** **(IQR)**	**Median** **(IQR)**	**Median** **(IQR)**	***p***	**Median** **(IQR)**	**Median** **(IQR)**	**Median** **(IQR)**	***p***	**Median [95% IC]**	***p***
**Primary outcome: ISI score**										
ISI score	17.00 (5.00)	16.00 (6.50)	3.00 (7.00)	0.009	19.50 (3.00)	12.00 (9.00)	9.00 (7.00)	<0.0001	−7.00 [−10.00;−3.00]	0.0004
**Secondary outcomes: Sleep diaries parameters**										
TIB (min)	489.46 (59.64)	512.32 (53.60)	−7.50 (37.71)	0.44	508.57 (47.86)	421.79 (89.40)	68.75 (80.96)	<0.0001	−67.20 [−95.66; −41.07]	<0.0001
TST (min)	289.29 (114.63)	319.64 (112.65)	−26.07 (89.29)	0.04	303.57 (113.21)	335.18 (61.07)	−53.04 (79.29)	0.002	+24.64 [−17.14; +66.79]	0.26
SE	0.61 (0.22)	0.68 (0.19)	−0.04 (0.18)	0.06	0.61 (0.17)	0.82 (0.17)	−0.18 (0.19)	<0.0001	+ 0.15 [+0.09; +0.23]	<0.0001
Sleep onset latency (min)	33.21 (31.79)	32.50 (22.14)	1.96 (25.12)	0.60	41.96 (55.25)	15.89 (26.07)	23.80 (33.57)	0.0008	−23.93 [−42.50; −9.29]	0.004
Number of awakenings	1.04 (0.80)	0.64 (0.86)	0.11 (0.79)	0.09	1.14 (1.00)	0.32 (0.64)	0.79 (1.14)	0.0003	−0.50 [−0.93; −0.07]	0.03
Wake after sleep onset (min)	36.61 (28.57)	32.86 (39.64)	8.04 (22.68)	0.10	35.36 (25.00)	9.64 (33.93)	22.68 (33.57)	0.02	−11.79 [−28.21; +6.43]	0.16
**Other outcomes: Self-report functional outcomes**										
ESS score	9.00 (6.00)	10.00 (8.00)	1.50 (5.00)	0.29	10.00 (6.00)	8.00 (4.00)	0.50 (4.00)	0.13	0.00 [−2.00; +2.00]	0.93
BDI score	14.50 (10.00)	8.00 (8.00)	5.00 (10.00)	0.02	14.50 (12.00)	5.00 (4.00)	6.50 (10.00)	<0.0001	−3.00 [−8.00; +2.00]	0.21
EQ-5D utility score	0.78 (0.12)	0.78 (0.09)	0.00 (0.05)	0.92	0.76 (0.09)	0.78 (0.09)	0.00 (0.09)	0.16	0.02 [0.00; +0.09]	0.21
EQ-5D VAS	60.00 (20.00)	61.00 (20.00)	−3.50 (22.00)	0.35	67.50 (20.00)	75.00 (10.00)	−15.00 (25.00)	0.004	+10.00 [−5.00; +25.00]	0.17
STAI-E	35.00 (14.00)	37.00 (16.00)	−0.50 (7.00)	0.79	35.00 (14.00)	32.00 (12.00)	7.50 (12.00)	0.02	−6.00 [−11.00;0.00]	0.03
CFS physical score	4.00 (3.00)	4.00 (4.00)	0.00 (2.00)	0.63	5.00 (2.00)	3.00 (4.00)	1.00 (2.00)	0.003	−1.50 [−3.00;0.00]	0.01

(1) *Indicates median change between baseline and post-treatment evaluation*.

(2) *Indicates median change difference between the mPT group and the eCBT-I group*.

**Table 3:** Change in Insomnia Severity Index from baseline in response to either online cognitive and behavioral therapy (eCBT-I) or minimal psychoeducation therapy (mPT) by participant characteristics in the mITT population.

	**mPT group**		**eCBT-I group**			
**Variable**	**No. of patients**	**Within-group ISI change from baseline^(2)^**	**No. of patients**	**Within-group ISI change from baseline^(2)^**	**Between-group difference ^(3)^**	***p*-value**
		**Median (IQR)**		**Median (IQR)**	**Median [95% CI]**	
**Age, years^(1)^**						
<45.5 years	10	1.00 (7.00)	8	10.50 (6.00)	−8.00 [−14.00; −2.00]	0.005
≥45.5 years	10	4.00 (5.00)	10	8.00 (11.00)	−5.00 [−10.00; 0.00]	0.07
**Gender**						
Male	6	4.00 (3.00)	3	13.00 (10.00)	−7.00 [−17.00; 7.00]	0.21
Female	14	2.00 (7.00)	15	8.00 (8.00)	−7.00 [−10.00; −3.00]	0.001
**ISI score^(1)^**						
<19	13	1.00 (7.00)	6	7.50 (8.00)	−6.00 [−12.00; 0.00]	0.02
≥19	7	5.00 (6.00)	12	9.50 (6.50)	−4.00 [−10.00; 1.00]	0.09
**BDI score^(1)^**						
<14	9	5.00 (2.00)	8	8.00 (7.50)	−3.00 [−10.00; 2.00]	0.12
≥14	11	1.00 (8.00)	10	11.00 (7.00)	−8.00 [−13.00; −4.00]	0.003

(1) *The cut-off values were based on the median values of the whole sample*.

(2) *Indicates median change between baseline and post-treatment evaluation*.

(3) *Indicates median change difference between the mPT group and the eCBT-I group*.

Significant changes between groups were also found for secondary nighttime outcomes, SE, TIB, SOL, NWAK between baseline and endpoint, with greater improvement in the eCBT-I group (Table 2). All parameters (i.e., TIB, TST, SE, SOL, WASO, and NWAK) improved in the eCBT-I group while only TST improved in the mPT group with similar tendency for SE.

Between-group differences were also significant for anxiety symptoms and fatigue, while depressive symptomatology improved in both groups, and perceived quality of life in the eCBT-I group only (Table 2). Daytime sleepiness levels remained stable along the study in both groups; however the frequency of EDS (ESS > 10) increased from 31.6 to 47.4% in the mPT group (*p* = 0.18), and decreased from 44.4 to 22.2% in the eCBT-I group (*p* = 0.05).

Most patients with CNS-active drug at baseline in the eCBT-I group decreased their hypnotic consumption (11/12, 91.7%) that includes eight patients who completely stopped the drugs during the study. In contrast, two out of 12 patients (16.7%) either reduced (one patient) or stopped (one patient) their medication in the mPT group.

Randomized participants from both groups did not report any treatment-emergent adverse effect.

## Discussion

In a small clinical population of adult patients with insomnia disorder, our randomized controlled study showed the efficacy of online automated CBT-I interventions. Even preliminary, this program seems effective in reducing insomnia severity and improving sleep efficiency, sleep duration, sleep onset latency, number of nighttime awakenings but also fatigue, depressive and anxiety-related symptoms as well as perceived quality of life. Our results also support the abilities of this Internet-delivered program to help CNS-active medication discontinuation in patients with insomnia disorder. Altogether, this eCBT-I confirms the extensive evidence-based results associated with several existing commercial CBT platforms such as Sleepio, SHUTi, and Restore. However, to our knowledge this is the first online CBT-I program in French-speaking language.

The reduction of insomnia severity assessed on ISI in the eCBT-I group was impressive, 43.9%, and 19.4% in the mPT group in the mITT population, a result in line with other online CBT-I interventions (13, 14). Data retrieved from the sleep diaries completed along the program also revealed a strong improvement in SE, SOL, and NWAK for patients following the eCBT-I program compared to patients assigned to the mPT group. These results were similar to those found in meta-analyses of automated CBT-I, and to those reported after either face-to-face or group delivered conventional CBT-I (13, 14, 34, 35).

We also reported that compared to the control group, fatigue, and anxiety symptoms improved in the eCBT-I group while a better quality of life assessed on a widely used visual analogic scale of the EQ5D was perceived in the eCBT-I group only. In contrast, depressive symptoms improved in both groups, a result in line with previous studies using eCBT-I programs showing reduction in affective symptoms even in the absence of specific interventions targeting depressive or anxiety symptoms (36). In contrast, no change in EDS complaint was found that may relate to a floor effect (rare patients with insomnia with high level of EDS) and sleep restriction instructions (37, 38). In contrast to most of sleep diary data, most of self-report functional parameters did not reveal between group differences from baseline to endpoint. These findings may relate to the nature of the control condition. Our control group was in fact an active group, as it consisted on a 1 h face-to-face psychoeducation that offers high-quality information about sleep physiology and insomnia. Some of these patients benefited to this educational program (i.e., −3.0 points on ISI score) that may be a confounding factor thus minimizing the observed global differences between eCBT-I and control groups. In contrast, most of RCT on eCBT-I typically used a passive waiting list as the control group with thus higher between-group differences expected (13, 14, 38).

Overall, the online CBT-I intervention was well-received by participants, without adverse effects. Dropout attrition (subjects not completing final evaluation measures) was similar to previous RCT on eCBT-I, with 21.74% of lost.

Our eCBT-I program might also help in tapering off hypnotic medication use (with more than 90% of patients allocated to eCBT-I reducing or stopping sleep aids), even in the absence of specific given instructions. Several studies have highlighted the benefit of behavioral interventions (stimulus control, sleep restriction, relaxation) within the context of gradual tapering of benzodiazepines among patients with chronic insomnia. Such interventions may attenuate withdrawal symptoms and prevent relapse up to 12-month follow-up (15, 16, 39). This unexpected outcome is of major interest for general and socio-economic public health perspectives since 30% of the general population in France occasionally use anxiolytics or hypnotics (with 5–7% chronic users), making French users two to three time superior to most industrialized countries (40, 41). Dissemination of online mental health services has increased in the recent years and may thus change the management of high prevalence disorders. Our results showed comparable efficacy on insomnia measures than face-to-face CBT-I delivered by psychologists. Altogether, these findings have important policy implications, and eCBT-I should be part of initiative to educate the public and to manage some patients with insomnia after a comprehensive diagnosis made by physicians (42).

Several limitations should be taken into consideration when interpreting our results. The sample size is really small; however it is a clinically-derived population with strict eligibility criteria (i.e., sleep efficiency ≤ 79% based on two-week sleep diary, an ISI score > 14). The exclusion of participants with clinically significant comorbid medical disorders or multiple psychiatric comorbidities may limit the generalizability of the results (43). Although the participants were recruited from a tertiary outpatient sleep center, the population included was well-educated and comfortable using the Internet. The post-treatment evaluation was conducted 2 weeks after the last intervention that preclude to assess on the long-term maintenance of efficacy of the eCBT-I. No long-term benefit-risk assessment was planned for this study. No objective measures of sleep such as polysomnography or actigraphy were used. However, the severity of insomnia complaints was assessed using the Insomnia Severity Index and sleep diaries, which are reliable and valid insomnia measures.

In conclusion, we developed the first eCBT-I program in French language and reported that this program delivered using online support seems feasible, acceptable and effective in improving sleep and related functional impairments in a small clinical population of adults with insomnia disorder but also in reducing CNS-active drug consumption. Our preliminary results should be replicated in larger, potentially less severe populations of patients with insomnia, and with multiple comorbid conditions to clarify if our findings are generalizable and maintained (44). Given the high prevalence of insomnia, the large use of Internet worldwide and the robust effects reported, our data are supportive of the use of eCBT-I as an effective alternative to treat insomnia in daily clinical practice in French speaking countries.

## Data Availability Statement

The datasets generated for this study are available on request to the corresponding author.

## Ethics Statement

The studies involving human participants were reviewed and approved by CPP Sud Méditerranée I, France, Number 2014-A01796-41. The patients/participants provided their written informed consent to participate in this study.

## Author Contributions

YD and RL: drafting/revising the manuscript for content, including medical writing for content, study concept or design, interpretation of data analysis, and study supervision and coordination. MB and A-DB: authors of the French version of eCBT-I. SB and IJ: revising the manuscript for content, interpretation of data analysis, study concept or design, and statistical analysis. EE, LB, SC, and AB: revising the manuscript for content, interpretation of data analysis, and acquisition of data.

### Conflict of Interest

RL has received funds for speaking and travel to conferences from UCB Pharma, Shire, and HAC. YD has received funds for speaking, board engagements, and travel to conferences from UCB Pharma, Jazz, Bioprojet, and Theranexus. AD-B is employee of Meta-Coaching Company who developed the Therasomnia program. TAKEDA and IDORSIA for YD. The remaining authors declare that the research was conducted in the absence of any commercial or financial relationships that could be construed as a potential conflict of interest.
